# Correcting *B*_0_ inhomogeneity-induced distortions in whole-body diffusion MRI of bone

**DOI:** 10.1038/s41598-021-04467-2

**Published:** 2022-01-07

**Authors:** Leonardino A. Digma, Christine H. Feng, Christopher C. Conlin, Ana E. Rodríguez-Soto, Allison Y. Zhong, Troy S. Hussain, Asona J. Lui, Kanha Batra, Aaron B. Simon, Roshan Karunamuni, Joshua Kuperman, Rebecca Rakow-Penner, Michael E. Hahn, Anders M. Dale, Tyler M. Seibert

**Affiliations:** 1 Department of Radiation Medicine and Applied Sciences, School of Medicine, University of California San Diego, 9500 Gilman Drive, Mail Code 0861, La Jolla, CA 92093-0861 USA; 2 Department of Radiology, School of Medicine, UC San Diego, 9500 Gilman Drive, La Jolla, CA 92093 USA; 3 Department of Electrical and Computer Engineering, UC San Diego, 9500 Gilman Drive, La Jolla, CA 92093 USA; 4 Department of Bioengineering, UC San Diego, 9500 Gilman Drive, La Jolla, CA 92093 USA

**Keywords:** Bone imaging, Magnetic resonance imaging, Whole body imaging

## Abstract

Diffusion-weighted magnetic resonance imaging (DWI) of the musculoskeletal system has various applications, including visualization of bone tumors. However, DWI acquired with echo-planar imaging is susceptible to distortions due to static magnetic field inhomogeneities. This study aimed to estimate spatial displacements of bone and to examine whether distortion corrected DWI images more accurately reflect underlying anatomy. Whole-body MRI data from 127 prostate cancer patients were analyzed. The reverse polarity gradient (RPG) technique was applied to DWI data to estimate voxel-level distortions and to produce a distortion corrected DWI dataset. First, an anatomic landmark analysis was conducted, in which corresponding vertebral landmarks on DWI and anatomic *T*_2_-weighted images were annotated. Changes in distance between DWI- and *T*_2_-defined landmarks (i.e., changes in error) after distortion correction were calculated. In secondary analyses, distortion estimates from RPG were used to assess spatial displacements of bone metastases. Lastly, changes in mutual information between DWI and *T*_2_-weighted images of bone metastases after distortion correction were calculated. Distortion correction reduced anatomic error of vertebral DWI up to 29 mm. Error reductions were consistent across subjects (Wilcoxon signed-rank p < 10^–20^). On average (± SD), participants’ largest error reduction was 11.8 mm (± 3.6). Mean (95% CI) displacement of bone lesions was 6.0 mm (95% CI 5.0–7.2); maximum displacement was 17.1 mm. Corrected diffusion images were more similar to structural MRI, as evidenced by consistent increases in mutual information (Wilcoxon signed-rank p < 10^–12^). These findings support the use of distortion correction techniques to improve localization of bone on DWI.

## Introduction

Diffusion-weighted magnetic resonance imaging (DWI) of the musculoskeletal system has wide-ranging applications, such as visualization of vertebral fractures, ligament tears, and assessment of bone quality with aging and osteoporosis^1,2^. Recently, DWI has been shown to also have utility in detecting and localizing bone tumors^3–5^. In each of these applications, DWI offers several advantages. In the context of bone tumors, for example, DWI has improved spatial resolution compared to the more commonly used technetium-99m skeletal scintigraphy. Furthermore, DWI does not expose patients to radiation, unlike bone scan, positron emission tomography, and computed tomography (PET/CT) scan, which are also frequently used to evaluate metastases.

Despite its advantages, DWI of bone suffers from technical challenges that limit its clinical adoption. One limitation is distortion induced by static magnetic field (*B*_0_) inhomogeneity. This artifact is inevitably present in single-shot echo-planar imaging (EPI) techniques used to collect DWI^6^ and can cause substantial warping of images. In image-guided clinical interventions where accurate localization is critical, such as stereotactic radiosurgery or biopsy, these distortions may be severe enough to impact clinical results. Moreover, DWI is also becoming increasingly utilized to evaluate treatment response of bone tumors^7–10^. Assessing response to medical or radiation treatment also demands highly accurate localization and measurement of lesion size, both of which may be affected by distortions induced by *B*_0_ inhomogeneity.

Several general approaches have now been developed to measure and correct for these artifacts^11–13^, but none has yet been widely adopted for routine clinical use. One approach attempts to reduce the extent of distortions prospectively by using a reduced field-of-view (FOV)^14^. This acquisition reduces the EPI readout time, which in turn limits the time allowed for the spin dephasing that leads to *B*_0_ inhomogeneity distortions. An alternative approach is correcting for the distortions retrospectively. This can be achieved by acquiring images at different echo times and quantifying the change in phase to produce a field map that is then used to correct the distortion artifact^13^. An additional retrospective approach involves leveraging the symmetry of distortions when EPI data are acquired with opposite phase encoding directions. One can acquire a diffusion volume in both the positive and negative phase encoding trajectory directions, and then estimate a displacement field from these images. The resulting field is then used to correct the diffusion data set. Both FSL’s topup and the reverse polarity gradient (RPG) technique are specific implementations of this approach^11,12,15^. The latter, RPG^12^, has been applied to DWI of prostate^16^, breast tissue^17,18^, and brain^12,19^, and has been shown to improve anatomic localization.

The magnitude of the *B*_0_ inhomogeneity-induced distortion that affects a particular region within the field of view is dependent on several factors, including proximity to tissue boundaries (e.g., air-tissue interfaces) and location relative to the scanner iso-center. Bone occupies anatomic environments (i.e., have different anatomic neighbors) that are distinct from that of prostate, breast, and brain. Thus, the impact of *B*_0_ inhomogeneity distortions on the spatial localization of bone or whole-body DWI is not known. In this study, we examined *B*_0_ inhomogeneities in whole-body DWI and investigated how they can affect visualization of bone anatomy and localization of bone metastases. We used RPG to estimate the magnitude of these distortions in images of bone and to determine whether correcting for these distortions would improve anatomic correlation of skeletal DWI with *T*_2_-weighted images. We also performed a secondary analysis to explore how these distortions affect localization of bone metastases.

## Methods

### Study population

Patients with suspected or known metastatic prostate cancer were enrolled in a prospective, observational, non-contrast whole-body MRI trial at the University of California San Diego from August 2017 to October 2020. The study was approved by the University of California San Diego Institutional Review Board (IRB #151686). All study participants were over the age of 18 years old and provided written informed consent. The study was done in accordance with the Declaration of Helsinki and Good Clinical Practice guidelines.

### Image acquisition and processing

Whole-body MRI was acquired on a 3.0 T MRI system (Discovery MR750, GE Healthcare, Waukesha, WI, USA) with a multi-parametric MRI protocol that included *T*_2_-weighted (TE/TR: 113/1350 ms, acquisition matrix: 384 × 224, resampled matrix: 512 × 512, FOV: 400 × 400 mm, slices: 46, slice thickness: 6 mm) and DWI (TE/TR: 75/4750 ms, acquisition matrix: 80 × 80, resampled matrix: 128 × 128, FOV: 400 × 400 mm, slices: 46, slice thickness: 6 mm, readout: single-refocused single-shot EPI, bandwidth: 3.9 kHz/pixel) sequences. The DWI protocol included diffusion weightings (*b*-values) of 0, 500, 1000, and 2000 s/mm^2^, with 1, 6, 6, and 12 diffusion directions (using the default tensor), respectively. Each subject underwent this protocol at 5 different stations to produce a whole-body image. This protocol did not use respiratory gating.

To estimate the distortion due to *B*_0_ inhomogeneity using RPG, *b* = 0 s/mm^2^ images were acquired in both the forward and reverse phase-encoded direction. The collection of the additional *b* = 0 s/mm^2^ image added 30 to 40 s to the protocol at each imaging station. The implementation details of the RPG algorithm has been described previously^12^ and was applied using in-house software. Briefly, the forward and reverse images are smoothed and registered with one another using a least-squares cost function. This registration is used to calculate an initial estimate of the distortion. This procedure is then repeated several times, and, in each iteration, the smoothing is performed with a thinner kernel and the distortion estimate is refined. The result of this procedure is a 3D distortion map where each value in the volume represents the number of voxels it was displaced in the phase encoding direction due to *B*_0_ inhomogeneity. This distortion map can be applied to the raw diffusion images for distortion correction (DisCo). *B*_0_-related distortion is independent of diffusion weighting, so this map can be used to correct the entire diffusion dataset to produce post-DisCo images.

For DWI b-value images that were collected in multiple directions (i.e., *b*-value images 500, 1000, 2000) volumes for each *b*-value were averaged prior to the analysis detailed below. All DWI volumes were also corrected for gradient non-linearity and eddy currents^20–23^.

### Statistical analysis

#### Anatomic landmark error reduction analysis

**Figure 1:**
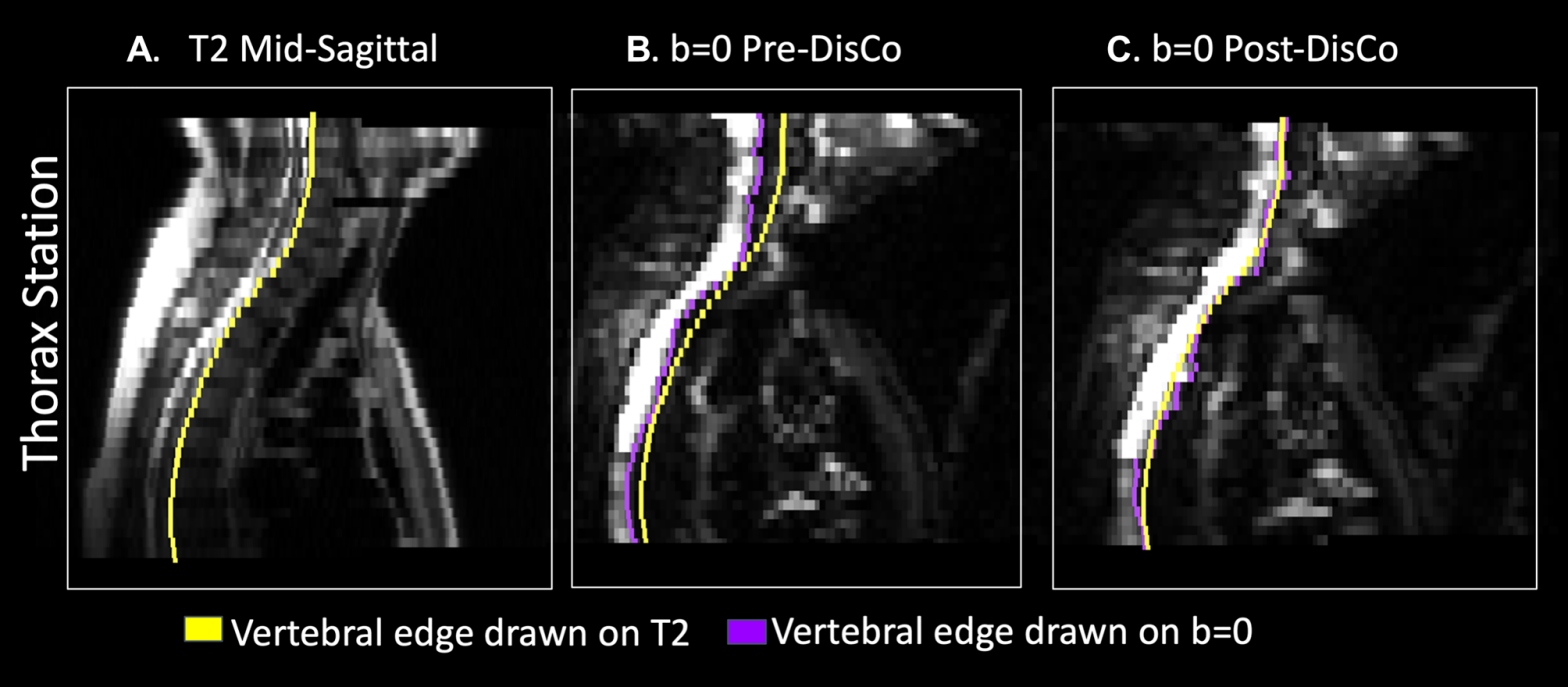
Example of tracing of posterior border of vertebrae for error change analysis. (**A**) Mid-sagittal slice of T2-weighted MRI from the Thorax Station for a single subject. The yellow represents the annotation of the posterior edge of the vertebral column on the T2. (**B**) Mid-sagittal slice of the DWI b = 0 s/mm^2^ prior to DisCo. The purple in this panel represents the annotation of the posterior edge of the vertebral column on the pre-DisCo DWI b = 0 s/mm^2^. (**C**) Mid-sagittal slice of DWI b = 0 s/mm^2^ after DisCo. The purple in this panel represents the annotation of the posterior edge of the vertebral column on the post-DisCo DWI b = 0 s/mm^2^. The average distance between the yellow and purple tracings (i.e., the error) decreases after application of DisCo. *DisCo* distortion correction.

We first performed an anatomic landmark analysis to estimate the extent to which DisCo could reduce anatomic error in DWI of bone. The posterior edge of the vertebral column in the mid-sagittal plane was selected as the landmark because it is present in multiple stations and is discernable on both DWI (*b* = 0) and *T*_2_-weighted images (the latter are less susceptible to distortion from *B*_0_ inhomogeneity). For each participant, we traced this landmark separately on the participant’s pre-DisCo *b* = 0, post-DisCo *b* = 0, and *T*_*2*_ images (for example, see Fig. 1). Then, at each axial slice, we calculated the distance (along the anterior–posterior axis) between the pre-DisCo *b* = 0 point annotation and the *T*_2_ point annotation as a measure of error. We further calculated the distance between the post-DisCo *b* = 0 point annotation and the *T*_2_ point annotation in order to calculate the change in error after DisCo. Each point error measure could have a different sign depending on whether the *b* = 0 point annotation erred on the anterior or posterior side of the T2 point annotation. In this analysis, we were simply focused on changes in the magnitude, rather than the direction, of error. Thus, we took the absolute value of each point error measurement before calculating the change in error. We recorded the largest error reduction measured for each patient and provided summary statistics for this distribution. We also calculated the mean error (across the length of the spine) for each patient and compared these mean errors pre- and post-DisCo with a Wilcoxon signed-rank test.

#### Estimating distortion of bone metastases

As discussed above, one practical application of DWI is its capability to detect bone metastases. Thus, we sought to characterize how distortions affected accurate localization of bone metastases in the present dataset. Bone metastases were identified based on available standard-of-care imaging (primarily CT, bone scan, and PET/CT) and DWI. These lesions were annotated manually on the diffusion images in MIM (MIM Software Inc, Cleveland, OH, USA) by a radiation oncologist (C.H.F.) with 4 years of experience. These annotations were then reviewed and confirmed by a fellowship-trained body radiologist (M.E.H.). Where applicable, the standard-of-care clinical imaging was also used to inform DWI lesion delineation. Only Stations 2, 3, and 4, were used in our analyses, as very few bone metastases were found in Stations 1 and 5. We refer to Stations 2, 3, and 4 as the Thorax, Abdomen, and Pelvis Station in the rest of this report.

**Figure 2:**
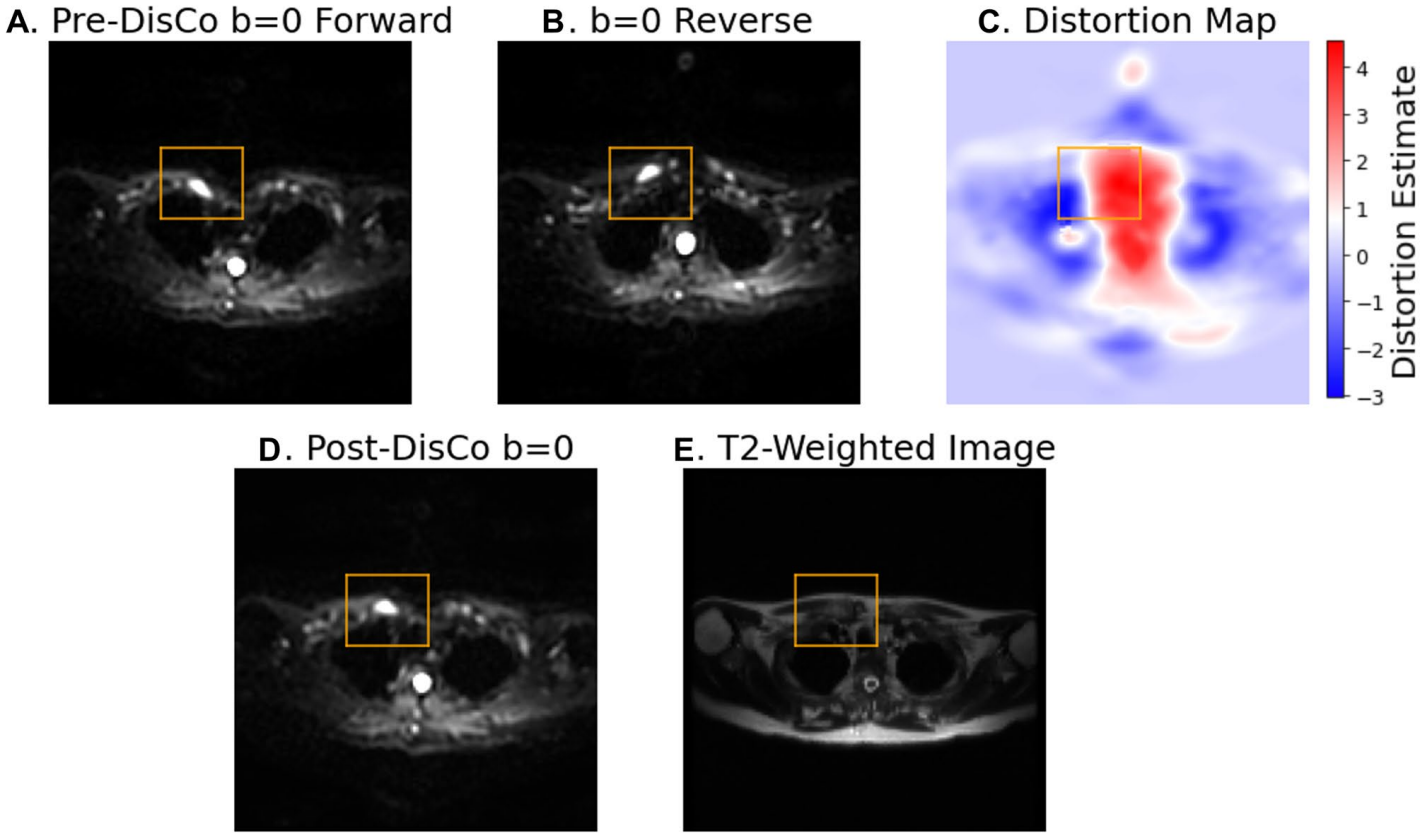
Example bone metastasis illustrating effect of B0 inhomogeneity induced distortion on DWI. In each subfigure, the slice corresponds to the horizontal slice at which the lesion is the largest (see “Methods” section). The orange bounding box was drawn 10 voxels from the lateral edge of the lesion (in DWI space) in each of the 4 directions, then overlaid on the other images. (**A**) DWI b = 0 s/mm^2^ image acquired in the forward phase encoded direction. (**B**) DWI b = 0 s/mm^2^ image acquired in the reverse phase encoded direction. (**C**) Estimation of the distortion within the slice. Voxel values represent the extent of displacement undergone by each voxel. Red and blue values denote displacement in the posterior and anterior direction, respectively. (**D**) DWI b = 0 s/mm^2^ image after DisCo. (**E**) T2-weighted image at the same slice. *DisCo* distortion correction.

For each lesion, we identified the horizontal slice in which the lesion was largest and selected it for analysis. Within that horizontal slice, a rectangular bounding box was drawn around each lesion, with the boundaries drawn 10 voxels from the lateral edge (in diffusion MRI space) of the lesion (for example, see Fig. 2). The bounding box was then overlaid onto the distortion map. To quantify the extent to which distortion occurred within the lesion and in the immediate surrounding area, the root mean square (RMS) within the box was calculated using the following formula, as described previously^16^:

1 $${RMS}_{distortion}=2 \times \sqrt{{\mu }_{distortion}^{2}+{\sigma }_{distortion}^{2}},$$

where ${\mu }_{distortion}$ and ${\sigma }_{distortion}$ represent the mean and standard deviation of the distortion values of the voxels within the bounding box, respectively. We performed bootstrapping to obtain a 95% confidence interval for the mean RMS. To this end, we generated bootstrap samples by subject-level re-sampling with replacement. 10,000 such samples were generated, and a mean RMS was calculated for each sample to generate a distribution of means. The 2.5th and 97.5th percentiles were taken as the 95% confidence interval for the mean RMS.

#### Assessing similarity between diffusion and structural images

The above analysis used DisCo to estimate the displacement of lesions in uncorrected images. Next, we conducted a mutual information analysis to assess the degree to which DisCo produces diffusion images that more faithfully reflect underlying anatomy of the *T*_2_-weighted images. The *T*_2_ image for each subject was resampled to match the resolution of the DWI images, using spline interpolation of order 3. For each lesion, we computed the normalized mutual information (MI) between the pre-DisCo *b* = 0 image and *T*_2_ image, and the normalized MI between the post-DisCo *b* = 0 image and *T*_2_ image. MI values were calculated for the entire horizontal slice^17^. We compared pre-DisCo and post-DisCo MI values with a Wilcoxon signed-rank test, with each lesion representing an observation. This Wilcoxon signed-rank analysis was then repeated for only the subset of lesions within each imaging station.

## Results

Our study included 127 participants with suspected bone metastases and complete whole-body multiparametric MRI data. 23 participants had visible bone metastases, with 75 individually annotated lesions across this subsample for inclusion in the bone lesion-level analysis.

### Anatomic landmark error reduction analysis

**Figure 3:**
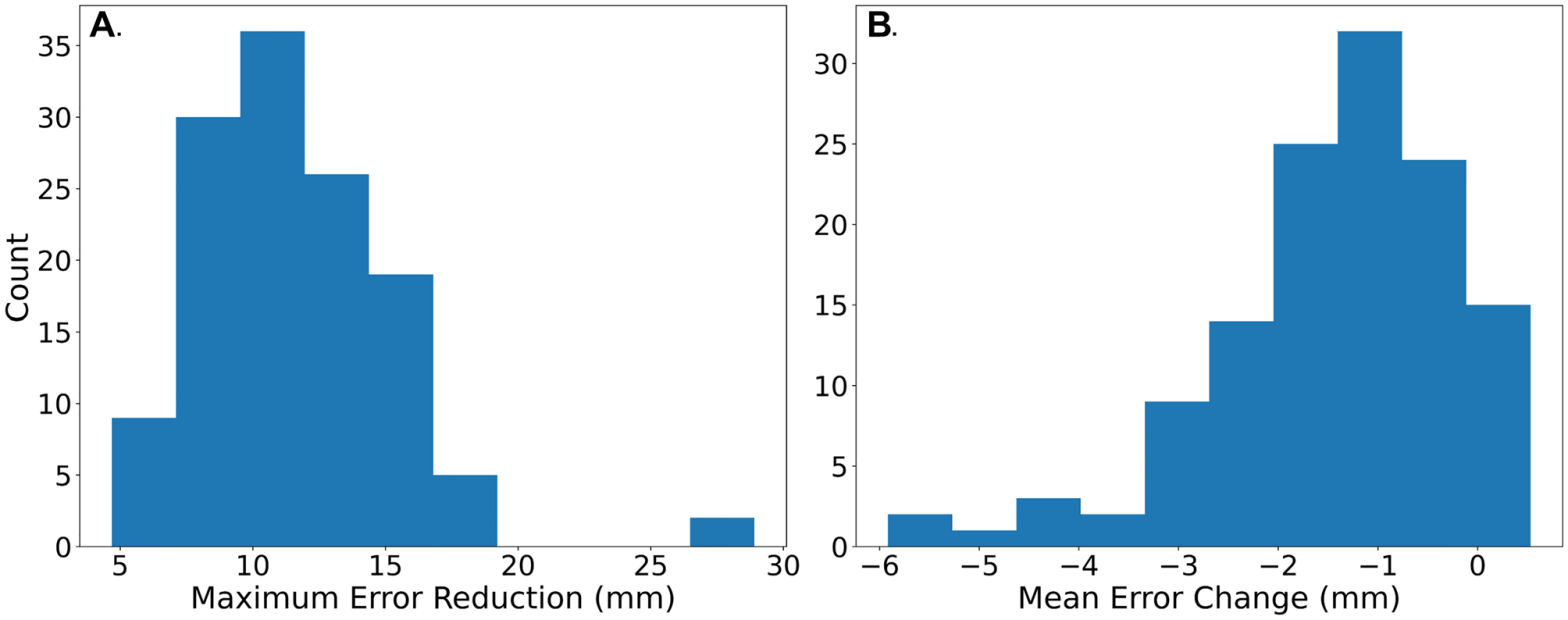
Application of DisCo leads to reduction in anatomic error of DWI. (**A**) Maximum error reduction was taken for each subject in our sample and plotted in a histogram. (**B**) Mean change in error before and after application of DisCo. *DisCo* distortion correction.

The mean (± SD) of largest error reduction (within each patient) was 11.8 mm (± 3.6 mm). The distribution of largest error reduction for each patient is shown in Fig. 3A. The largest observed error reduction was 29 mm, at a vertebral landmark in the thoracic vertebrae. Distortion correction led to consistent decreases in mean error for each subject (Wilcoxon signed-rank p < 10^–20^) (Fig. 3).

### Estimating distortion of bone metastases

**Figure 4:**
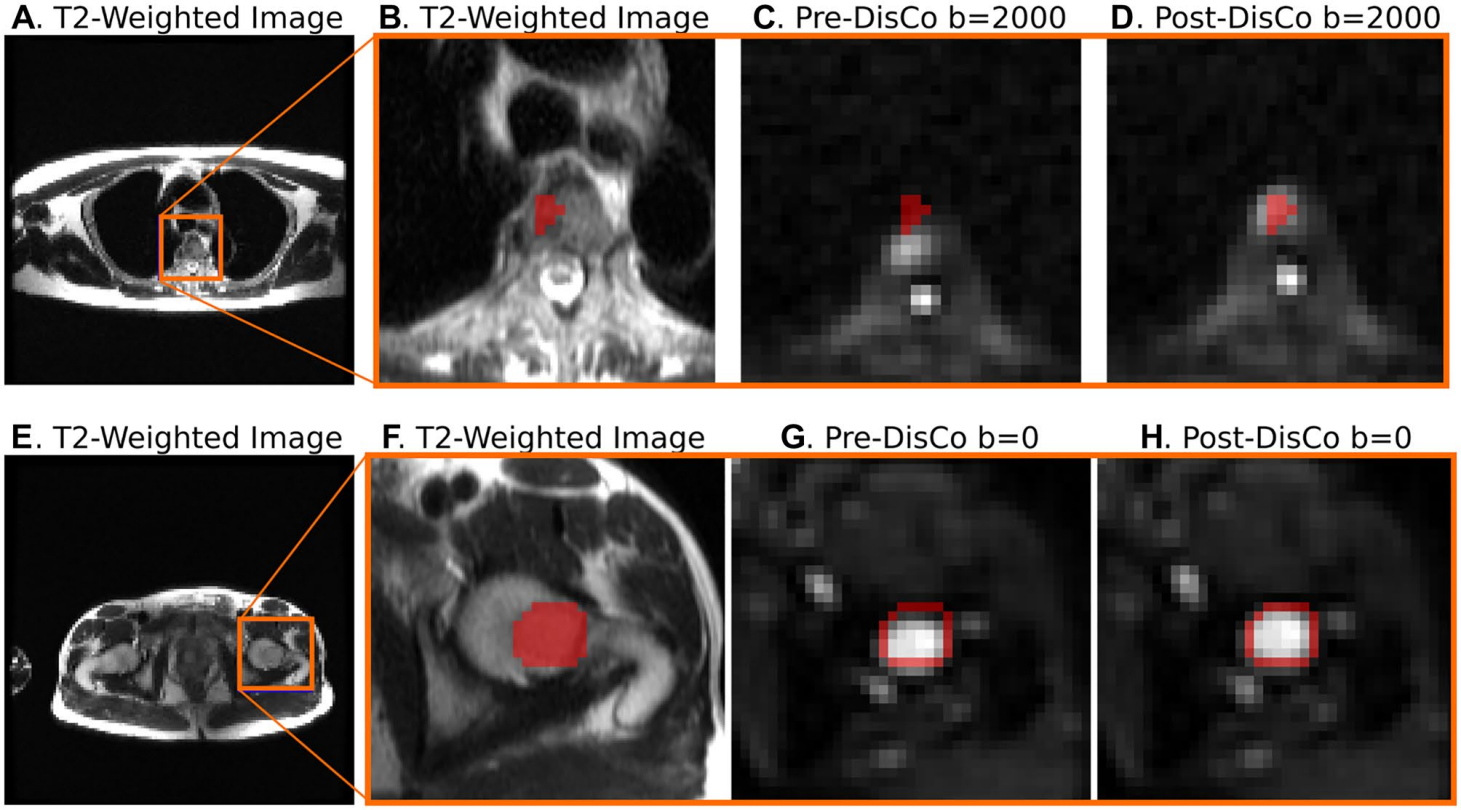
Examples of bone metastases as seen on b = 0 s/mm^2^ DWI pre-and post-DisCo. The top row (**A–D**) shows a vertebral lesion in the Thorax Station. The bottom row (**E–H**) shows a lesion within the left femoral head in the Pelvis Station. (**A,E**) T2-weighted images at the corresponding level with an orange bounding box drawn around the lesion. (**B,F**) T2-weighted images zoomed in to visualize lesion and surrounding area. (**C,G**) Zoomed in DWI b = 2000 s/mm^2^ and b = 0 s/mm^2^ images before DisCo, respectively. (**D,H**) Zoomed in DWI b = 2000 s/mm^2^ and b = 0 s/mm^2^ images after DisCo, respectively. Outlines of the lesion annotations are overlaid in red. In both examples, the lesions were translated anteriorly following DisCo. Without DisCo, lesion localization may have erred posteriorly. *DisCo* distortion correction.

**Figure 5:**
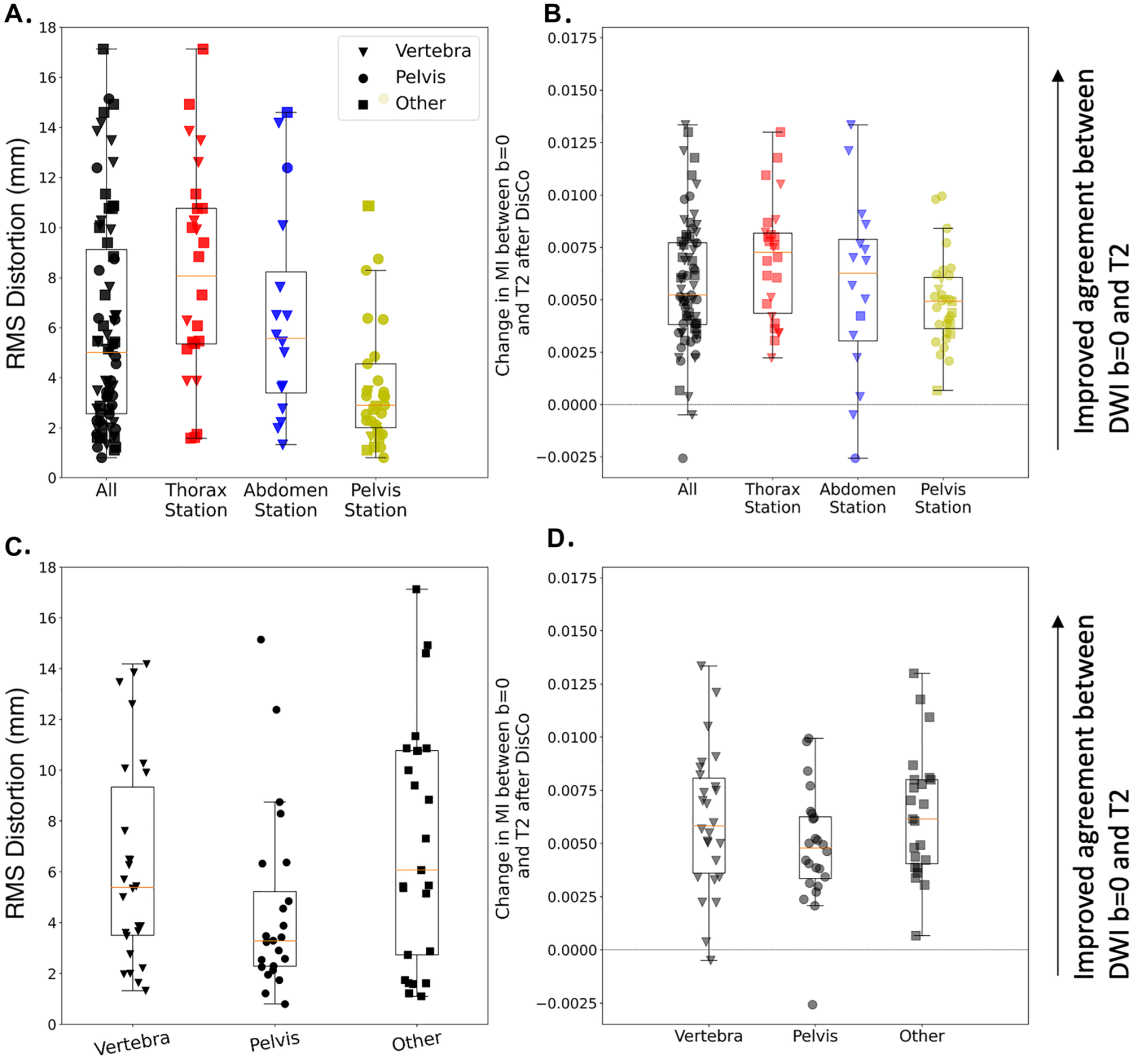
Distribution of RMS of distortion and MI between b = 0 s/mm^2^ and T2-weighted images. (**A**) Distortion for lesions across all stations (black) as well as the distribution within specific imaging Stations (red, blue, and yellow). Distortion was estimated by calculating the RMS of the values in the distortion map that correspond to the lesion annotation and immediately surrounding areas (see Eq. (1)). (**B**) Change in MI values between the DWI b = 0 s/mm^2^ and T2-weighted images after DisCo. A value larger than 0 indicates improved agreement between DWI b = 0 s/mm^2^ and T2-weighted images. Again, we plot the distribution for all lesions as well as the breakdown for different Stations. (**C**) For illustrative purposes, we categorized lesions into three distinct anatomic groups: Vertebra, Pelvis, and Other. The Other category represents lesions in bones such as the clavicle, sternum, and femur. The distribution for distortions for each anatomic group is plotted. (**D**) Change in MI values between the DWI b = 0 s/mm^2^ and T2-weighted images after DisCo broken down by anatomic group. DisCo led to consistent increases in similarity between DWI b = 0 s/mm^2^ and T2-weighted images. *RMS* root mean square, *MI* mutual information.

The maximum RMS was 17.1 mm and was observed in a clavicular lesion in the Thorax Station. In the Abdomen and Pelvis Station, the maximum RMS values were 14.6 mm and 15.1 mm, respectively. Across all lesions, the average RMS was 6.0 mm (95% CI 5.0–7.2). When grouped by station, the mean RMS for Thorax, Abdomen, and Pelvis Stations were 8.2 mm (95% CI 6.6–10.6), 6.5 mm (95% CI 4.3–8.5), and 4.1 mm (95% CI 2.7–5.9), respectively. Examples of vertebral and femoral lesions are displayed in Fig. 4. The distributions of RMS values are illustrated in Fig. 5. RMS values did not significantly differ when using bounding boxes with edges at 7 or 12 voxels away from the lesion edges.

To explore the extent to which *B*_0_ inhomogeneity may induce geometric distortions, we plotted example lesions from different parts of the skeleton and in different imaging stations (Figs. 2, 4). The metastasis in the left femoral head shown in Fig. 4E–H, for example, underwent *B*_0_ inhomogeneity-induced contraction, as can be appreciated with the dark strip of voxels at the anterior edge of the lesion in Fig. 4G. Additionally, the right clavicular lesion included in Fig. 2 became more globular in shape and was partially rotated after application of DisCo.

### Assessing similarity between diffusion and structural images

Applying DisCo led to consistently increased MI between the DWI *b* = 0 s/mm^2^ and *T*_2_ images. The average (± SD) change in MI between pre-DisCo and post-DisCo was 0.0057 (± 0.0030). When broken down by station, the average (± SD) changes in MI were 0.0069 (± 0.0028), 0.0056 (± 0.0042), and 0.0049 (± 0.0021), for the Thorax, Abdomen, and Pelvic Stations, respectively. When comparing pre-DisCo to post-DisCo MI values with Wilcoxon signed-rank tests, we found that the post-DisCo MI values were significantly higher (p < 10^–12^), indicating the RPG distortion correction improved similarity of DWI with the anatomic *T*_2_ images (Fig. 5). This was consistent for each of the imaging stations analyzed (Thorax Station: p < 10^–5^, Abdomen Station: p = 0.0004, Pelvis Station: p < 10^–5^).

## Discussion

Application of RPG to DWI led to consistent improvement in the anatomic accuracy of DWI of the vertebral skeleton. We found reductions in anatomic inaccuracy of up to 29 mm using RPG. On average, the participants’ largest error reduction was 11.8 mm (± 3.6). Similarly, for bone metastases, we observed a mean displacement of 6.0 mm (95% CI 5.0–7.2) and displacement of up to 17 mm, which are meaningful distortions in potential clinical applications like image-guided biopsy (where sampling the more hypercellular part of the lesion may be desired) or image-guided treatments like stereotactic radiotherapy^24^. As DWI continues to take on an increasing role in musculoskeletal imaging^1^, distortion correction may facilitate improved localization of bone tissue and associated pathology, especially in applications where high precision is required.

Prior studies have demonstrated that *B*_0_ inhomogeneities affect organ or lesion localization in the prostate and breast^16,17,18^. The extent of *B*_0_ inhomogeneities and their consequent artifacts depend, in part, on the anatomic environment of a region of interest or lesion (e.g., how close the lesion is to an air-tissue interface). Since bone occupies anatomic environments distinct from those of prostate and breast, it remains to be determined whether and to what extent these artifacts affect DWI of bone. Our findings here suggest that, like prostate and breast, bone is also susceptible to meaningful distortion.

Estimated distortion of lesions from *B*_0_ inhomogeneity was greatest in the Thorax Station and smallest in the Pelvis Station. The elevated RMS values in the Thorax Station, relative to the other imaging stations, may be due to a number of factors. As noted above, the magnitude of *B*_0_ inhomogeneities and their consequent artifacts depend partly on proximity to an air-tissue interface. The Thorax Station images several bones close to air–tissue interfaces, including the clavicles, ribs, and scapulae. Since these bones are all close to air–tissue interfaces, there are several places within the FOV of the Thorax Station where bone and bone metastases may be prone to heavy distortion. However, the clavicles, ribs, and sternum are also subject to respiratory motion, and we are not able to separate these effects in this study. Future studies might benefit from controlling for respiratory motion, such as abdominal compression, active breathing control, or respiratory gating^25^. Nevertheless, we found that the vertebrae of the Thorax Station, which are less affected by respiratory motion, still exhibited elevated RMS suggesting that not all of the distortion observed in the Thorax Station in our study can be explained by respiratory motion.

In addition to the spatial displacements that we have demonstrated in the skeletal landmark and bone lesion analyses, *B*_0_ inhomogeneities can also lead to geometric distortions. Voxels within a diffusion volume can undergo varying levels of displacement along the phase-encoding gradient. Fluctuating amounts of distortion within and around bone can lead to contraction, expansion, or other geometric distortion of the bone tissue as demonstrated in our example lesion in the femoral head. One clinical application where geometric distortion may cause concern is in the tracking of treatment response of bone metastases, where DWI has demonstrated utility^7,26^. Artificial geometric distortions from *B*_0_ inhomogeneity like those demonstrated in Figs. 2 and 4 could lead to misinterpretations in patients’ responses to therapy, such as mistaking imaging-related contraction or expansion distortions for tumor shrinkage or growth.

There are several tools available to correct for these artifacts and reduce the likelihood of missing a target or misinterpreting treatment response^11–13^. In the RPG method used here, a correction that can be applied to the diffusion dataset is simultaneously generated while calculating the voxel-wise displacements; this method was chosen for its efficiency, as little additional scan time (30–40 s per station) was required. A salient question, however, is whether this correction actually produces images that more accurately reflect anatomy. We demonstrated consistent reductions in mean error across subjects, as well as the increases in MI between DWI *b* = 0 and *T*_2_ images after correcting for distortion. This is in line with prior work that demonstrated improved similarity of DWI with *T*_2_ anatomic imaging after RPG^17,18^. Inspection of data revealed some instances of increases in measured error after the application of RPG. These may be due to actual movement of the patient or internal organs (e.g., via breathing or peristalsis) between DWI and *T*_2_ acquisitions. Nevertheless, the net effect of RPG was to consistently reduce measured errors. Taken together, our findings suggest that the RPG method is capable of generating diffusion images that more closely represent the true anatomy.

Limitations of our study include the potential effects of respiration on our distortion estimates that were discussed above. Beyond respiratory motion alone, slight differences in respiratory volume could also lead to artifacts. Thus, even if image acquisition were gated with the respiratory cycle, it is likely that some residual distortion would remain. Our group is actively working on imaging protocols and processing methods to address this residual distortion, which is relevant not only to imaging of bones near the lungs, but also to imaging of other organs near the diaphragm, such as the liver and pancreas. We also note that our whole-body imaging protocol does not capture the bones of the distal extremities. In the case of cancer, metastases in the distal extremities are uncommon. In other settings of musculoskeletal DWI, however, such as knee imaging, these locations are of interest. A larger-bore system might allow inclusion within the field of view. Future studies dedicated to characterizing the distortion in these anatomic locations will be needed as the artifacts will likely exhibit different characteristics. Another practical limitation is the added scan time to acquire the *b* = 0 volume in the reverse phase encoded direction. This was 30–40 s per station in our study. However, we have since worked with vendors to integrate the acquisition of the reverse *b* = 0 image as part of the same series in which the other diffusion volumes are collected; incorporating RPG now requires only a single addition repetition time (TR) per station—slightly less than 5 s per station. Further, given that RPG is a retrospective approach to distortion correction, it requires implementation of complex software to perform the necessary data post-processing. As previously discussed, FSL’s topup is another retrospective distortion correction approach that is similar in spirit to RPG^11,15^ and may afford another means of correcting these distortions. A final limitation is that our study sample only consisted of prostate cancer patients. Bone is also a common site of metastases in other cancers, particularly lung and breast cancer. DWI has been successfully used to visualize bone metastases in other cancers (for breast see^7^; for melanoma, see^27^). Lesion appearance may vary on DWI by cancer type, but there is no a priori reason to suspect that distortion effects would be substantially affected by cancer histology.

In summary, we found that *B*_0_ inhomogeneity results in distortion of whole-body diffusion images that leads to artifactual displacement of bone and bone metastases. These distortions may be severe enough to interfere with accurate biopsy or stereotactic treatment. Moreover, these distortions could complicate interpretation of tumor shrinkage or growth. The RPG technique used here is a highly efficient solution for reducing distortion artifacts in whole-body DWI.
